# Are natural deep eutectic solvents always a sustainable option? A bioassay-based study

**DOI:** 10.1007/s11356-022-23362-5

**Published:** 2022-10-04

**Authors:** Matilde Vieira Sanches, Rosa Freitas, Matteo Oliva, Angelica Mero, Lucia De Marchi, Alessia Cuccaro, Giorgia Fumagalli, Andrea Mezzetta, Greta Colombo Dugoni, Monica Ferro, Andrea Mele, Lorenzo Guazzelli, Carlo Pretti

**Affiliations:** 1 Department of Biology & Centre for Environmental and Marine Studies (CESAM), University of Aveiro, 3810-193 Aveiro, Portugal; 2 Interuniversitary Consortium of Marine Biology and Applied Ecology of Leghorn “G. Bacci”, 57128 Leghorn, Italy; 3 Department of Pharmacy, University of Pisa, 56126 Pisa, Italy; 4 Department of Chemistry, Materials and Chemical Engineering “G. Natta, Politecnico Di Milano, Piazza Leonardo da Vinci, 32, Milano, Italy; 5 Department of Veterinary Sciences, University of Pisa, San Piero a Grado, 56122 Pisa, Italy

**Keywords:** Eutrophication, Microalgae, Marine water, Freshwater, pH-dependant effects

## Abstract

The traditional use of organic solvents in various branches of industry is being rethought as these compounds very often display high volatility, toxicity and lipophilicity (related to the ability to interact with biological membranes). More recently, developments in the field of Green Chemistry are focusing on the design of more sustainable and cost-effective solvent alternatives like Ionic Liquids (ILs), bio-based solvents and natural deep eutectic solvents (NADESs). The present study aimed at performing an ecotoxicological screening of 15 NADESs using an extensive set of marine and freshwater bioassays, based on different endpoints as the following: immobilization of the crustacean *Daphnia magna*, growth inhibition of *Raphidocelis subcapitata* and of *Phaeodactylum tricornutum*, larval development alterations on the serpulid *Ficopomatus enigmaticus* and bioluminescence inhibition of *Aliivibrio fischeri*. What emerged was a general absence of toxicity of all samples. However, both algal assays showed a certain degree of biostimulation, up to over 100% growth increase in respect to controls with 8 out of 15 compounds tested with *Raphidocelis subcapitata*. Despite NADESs-induced negligible toxicity effects to invertebrates, encouraging their labelling as “sustainable” solvents, the liability of their intentional or accidental release into aquatic systems may represent a serious risk in terms of ecosystem functioning impairments.

**Supplementary Information:**

The online version contains supplementary material available at 10.1007/s11356-022-23362-5.

## Introduction

The traditional use of organic solvents in various branches of industry is being rethought as these compounds very often display high volatility, toxicity and lipophilicity, the latter usually related to the ability of such substances to interact with biological membranes (Singh et al. 2021). More recently, developments in the field of Green Chemistry are focusing on the design of more sustainable and cost-effective solvent alternatives like Ionic Liquids (ILs), bio-based solvents and Deep Eutectic Solvents (DESs) (Matzke et al. 2010; Domínguez de María 2017).

DESs may often be represented by mixtures of hydrogen bond acceptors (HBAs) and hydrogen bond donors (HBDs) which, when combined, become liquid at room temperature and result in a final solvent exhibiting a much lower melting point at the eutectic than what is expected for the ideal behaviour of the liquid mixture (Martins et al. 2019). Natural DESs (NADESs) can be prepared from primary metabolites. These primary metabolites are usually cholinium derivatives, sugars, amino acids and organic acids (Choi et al. 2011). Low vapour pressure when compared to traditional organic solvents, good chemical and thermal stability, low melting point and non-flammability are properties common to both ILs and DESs, with the latter ones being often suggested as potential substitute for the first ones (Chang et al. 2021). Such is due to the fact that, in comparison to ILs, besides being derived from renewable sources, DESs are claimed as non-toxic, biodegradable and cheaper to produce (Morais et al. 2015; Zdanowicz et al. 2018). Regarding the production process, large amounts of DESs can be synthesized resulting in little or no waste at all (Zhang et al. 2012; Singh et al. 2021).

The preparation of DESs comes as rather simple. Multiple steps or separation processes are not involved, and neither is the use of organic solvents (Singh et al. 2021). DESs’ starting components are usually inexpensive, renewably sourced and are obtained separately for preparation of the mixture (Cañadas et al. 2020). The synthesis of DESs relies on vacuum evaporation, freeze-drying, grinding and heating and stirring methods. This latter is the most commonly applied method and consists of heating and stirring DESs’ components, in an inert environment, until a homogeneous liquid is obtained and NADESs are prepared in a similar way (Singh et al. 2021). Nowadays, DESs are being applied to solubilisation of lignocellulosic biopolymers (Colombo Dugoni et al. (2020); Yiin et al. (2021); Marks and Viell (2022)), extraction of natural products (González-Rivera et al. (2021); Husanu et al. (2020); Cao et al. (2020)), solvents and catalysts for organic synthesis (Dilauro et al. (2021); Cicco et al. (2021)), pharmaceutical application (Silva et al. (2020)), electroanalysis (Arnaboldi et al. (2021)), metal electrodeposition (Rosoiu et al. (2021)), to name a few examples.

However, it is mandatory to be careful when terming DESs as sustainable. In fact, to date, information about their toxicological profile and biodegradability is still scarce (Lapeña et al. 2021). There are very few studies assessing the toxicity of DESs in aquatic invertebrates (Lapeña et al. 2021). The most represented species seems to be the marine bioluminescent bacteria *Aliivibrio fischeri* (Morais et al. 2015; Macário et al. 2018; Lapeña et al. 2021). Published studies have mainly focused on cellular lines and gram-positive and gram-negative bacteria (Hayyan et al. 2013a, b; Radošević et al. 2015; Torregrosa-Crespo et al. 2020). Generally, main results report that DESs display low toxicity and that some of these DESs are readily biodegradable.

The present study aimed at performing an ecotoxicological screening of 15 NADESs compounds by testing a wider variety of species than the one currently presented in the literature. Through the performance of an extensive set of bioassays, which aimed at different endpoints, the exposure effects of selected NADESs were assessed on the freshwater *Daphnia magna* (crustacean), *Raphidocelis subcapitata* (algae), and on the marine organisms *Phaeodactylum tricornutum* (algae), *Ficopomatus enigmaticus* (polychaete) and *Aliivibrio fischeri* (bacteria).

## Materials and methods

### Natural deep eutectic solvents (NADESs)

All chemical substances for NADESs preparation (betaine, proline, cholinium bitartrate, choline acetate (hydrogen bond acceptors — HBAs), ethylene glycol, citric acid, glycerol, levulinic acid, L-lactic acid, lactic acid, malic acid, imidazole, glycolic acid, diglycolic acid (hydrogen bond donors — HBDs) were purchased from Alfa Aesar GmbH. Purity of these chemicals was > 99%. NADESs synthesis followed the methodologies described by Hayyan et al. (2013b) and Colombo Dugoni et al (2020). Table 1 illustrates composition and molar ratios of the NADESs assessed in this study.

**Table 1:**
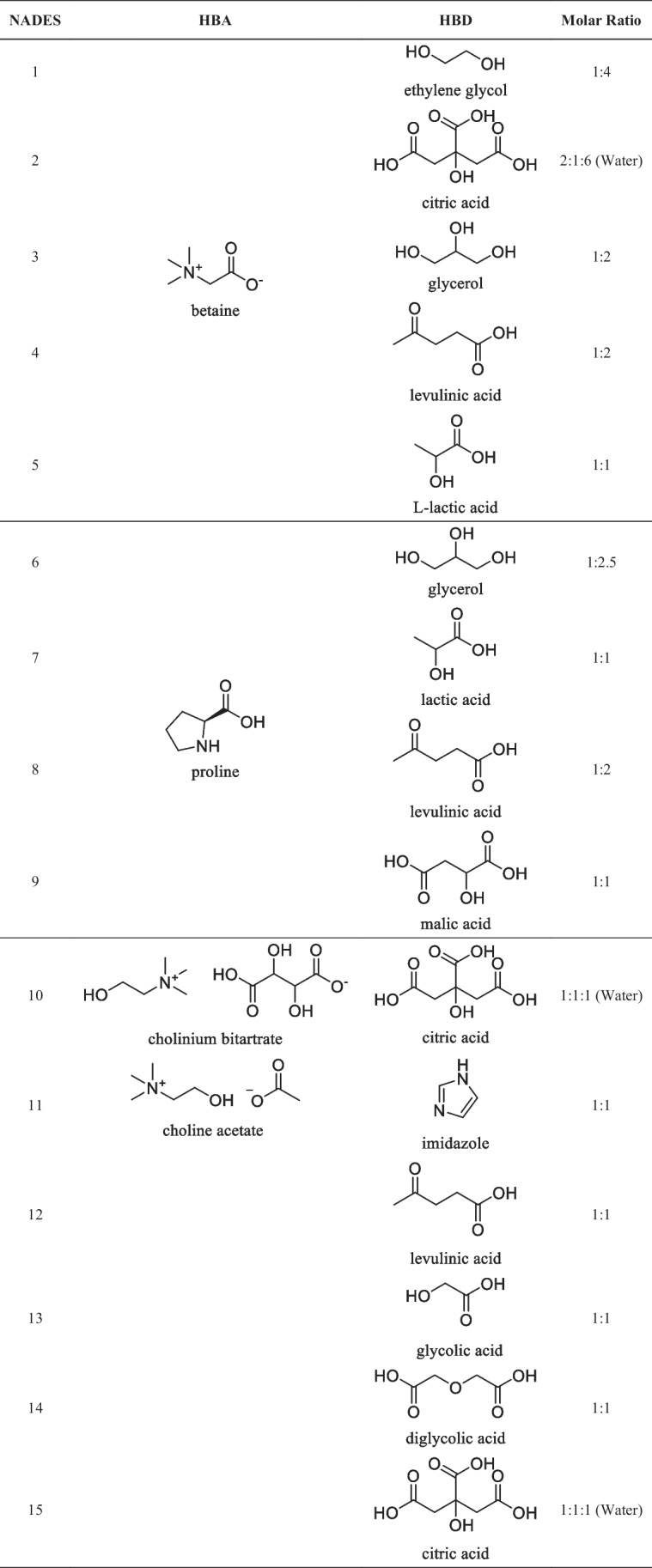
Structure of NADESs prepared in this study

### Stock and working solutions

Initial stock solutions were prepared in two media: artificial freshwater (AFW), according to ISO (2016a), and in artificial seawater (ASW), by the guidelines of ISO (2016b). For all tested NADESs, a screening concentration of 100 mg L^−1^ was prepared and, only after any significant effect detection, a dilution series was prepared at the following concentrations: 1, 2.5, 5, 10, 25, 50, 75, 100 mg L^−1^.

The pH of all 100 mg L^−1^ solutions was measured and reported in Tables A and B (Supplementary information).

### Ecotoxicological assessment

#### Daphnia magna — immobilization

The acute toxicity test with freshwater crustacean *D*. *magna* was performed according to the guidelines of ISO (2012). Ten organisms per replicate (nº = 3) were exposed to maximum concentration samples (100 mg L^−1^) of each NADESs for a period of 48 h, after which the number of immobile individuals was evaluated (no of immobilized individuals). Experiment conditions were 25 ± 2 °C, in darkness. One control lacking the test substance was run along the other samples. As instructed by the protocol, potassium dichromate (K_2_Cr_2_O_7_) was tested as reference toxicant — EC_50_ 0.586 mg L^−1^ (0.401–0.772).

#### Raphidocelis subcapitata — growth inhibition

Growth inhibition of the freshwater alga *R*. *subcapitata* was determined by the procedure ASTM (2012). *R*. *subcapitata* algae were cultured in Bold Basal Medium with threefold nitrogen and vitamins (3-N-BBM + V) and working batches were prepared by inoculating 2 mL of algal culture in 20 mL of fresh 3-N-BBM + V medium (20 ± 2 °C, under continuous illumination (6000–8000 lx)). After 72 h, algal batches were diluted to reach a concentration of 10^6^ cell mL^−1^. For the growth inhibition bioassay, algae, in 3 replicates, were exposed to maximum concentration NADES samples (100 mg L^−1^) for a period of 72 h, at 20 ± 2 °C and, once again, under continuous illumination (6000–8000 lx). The algal concentration, as cells mL^−1^, was calculated from spectrophotometrical absorbance measurement (Jenway Genova Plus), at wavelength = 670 nm, using the following equation, previously defined by the CIBM (Livorno, Italy) research group:

$${\varvec{C}}{\varvec{e}}{\varvec{l}}{\varvec{l}}{\varvec{s}}\boldsymbol{*}{{\varvec{m}}{\varvec{L}}}^{-1}=\frac{{{\varvec{A}}{\varvec{b}}{\varvec{s}}}_{670}}{{8\boldsymbol{*}10}^{-8}}$$

After calculation of algal concentration, percentage of growth difference (ΔG%) between samples and control were calculated as following:

$$\boldsymbol{\Delta }{\varvec{G}}\boldsymbol{\%}=\left(\frac{\left({\varvec{m}}{\varvec{e}}{\varvec{a}}{\varvec{n}}\boldsymbol{ }{\varvec{S}}\boldsymbol{*}100\right)}{{\varvec{m}}{\varvec{e}}{\varvec{a}}{\varvec{n}}\boldsymbol{ }{\varvec{C}}}\right)-100$$

where: “***mean S***” is the mean algal concentration in samples and “***mean C***” is the mean algal concentration in control.

After this screening test, samples which showed a ΔG% > 40% (biostimulation) in respect to control (Table A — Supplementary information) have been retested at concentrations from 1 to 100 mg L^−1^. All concentrations were prepared in double starting from 100 mg L^−1^ solution: one set with unadjusted pH (unadj-pH), and the other one with pH adjusted to control value (8.10) (adj-pH), by addition of few drops of 1 M NaOH solution.

Potassium dichromate was used as reference toxicant — EC_50_ 0.742 mg L-1 (0.648–0.808). For statistical analysis, a Student’s *t*-test was performed between (1) each concentration of unadj-pH and control; (2) adj-pH and control and (3) each concentration of unadj-pH and adj-pH.

Before screening test of NADES (maximum concentration, no pH correction), also a screening on single components was performed, in order to evaluate their effects in terms of algal growth inhibition/stimulation. What emerged was a negligible effect of each single component, with the exception of choline acetate, which showed to stimulate algal growth starting from a concentration of about 12.5 mg/L (data not shown).

#### Aliivibrio fischeri — inhibition of bioluminescence

Tests with the bioluminescent bacteria *A*. *fischeri* were carried out following the ISO (2007) methodology, for both freshwater and marine water samples. *A*. *fischeri* (strain n. 19A4002A, Ecotox LDS, Pregnana Milanese, MI, Italy) was purchased as freeze-dried bacterial cells. In a next step, these dried bacteria were resuspended in 1 mL of Reconstitution Solution in order to be reactivated. Regarding the assays, *A*. *fischeri* was exposed to 90% (marine protocol) or 81.9% (freshwater protocol) of maximum concentration samples (100 mg L^−1^) for 15 and 30 min, at a temperature condition of 15 ± 1 °C (2 replicates, pH: 6–8). Measurements were made in terms of differences in bioluminescence emission, expressed as percentage of bioluminescence inhibition (I %). According to the followed protocol (ISO 2007), the reference toxicant was zinc sulfate eptahydrate with EC_50_s of 1.04 mg L^−1^ (0.80–1.36) and 10.26 mg L^−1^ (9.22–11.43) Zn^2+^, for freshwater and seawater, respectively.

#### Phaeodactylum tricornutum — growth inhibition

Performing slight changes to the base protocol, the growth inhibition on the marine algae *P*. *tricornutum* was evaluated following ISO (2016b). *P*. *tricornutum* Bholin (CCAP 1052/1A) was the test strain used, purchased from the reference center CCAP (Culture Collection of Algae and Protozoa—Scottish Association for Marine Science/SAMS Research Services Ltd). *P*. *tricornutum* algae were cultured in ASTM Enriched Saltwater Medium (ASTM-ESM, ASTM 2012) and working batches were prepared by inoculating 2 mL of algal culture in 20 mL of fresh ASTM-medium (20 ± 2 °C, under continuous illumination (6000–8000 lx)). After 72 h, algal batches were diluted to reach a concentration of 10^6^ cell mL^−1^. For the growth inhibition bioassay, algae, in 3 replicates, were exposed to maximum concentration samples (100 mg L^−1^) for a period of 72 h, at 20 ± 2 °C and, once again, under continuous illumination (6000–8000 lx). Absorbance, at 670 nm, was measured in each well with a spectrophotometer, making use of 1 cm optic-path plastic cuvettes. The algal concentration, as cells mL^−1^, was calculated from absorbance values using the following equation, previously defined by the CIBM (Livorno, Italy) research group:

$${\varvec{C}}{\varvec{e}}{\varvec{l}}{\varvec{l}}{\varvec{s}}\boldsymbol{*}{{\varvec{m}}{\varvec{L}}}^{-1}=\frac{{{\varvec{A}}{\varvec{b}}{\varvec{s}}}_{670}}{{10}^{-7}}$$

After calculation of algal concentration, percentage of growth difference (ΔG%) between samples and control were calculated as the following:

$$\boldsymbol{\Delta }{\varvec{G}}\boldsymbol{\%}=\left(\frac{\left({\varvec{m}}{\varvec{e}}{\varvec{a}}{\varvec{n}}\boldsymbol{ }{\varvec{S}}\boldsymbol{*}100\right)}{{\varvec{m}}{\varvec{e}}{\varvec{a}}{\varvec{n}}\boldsymbol{ }{\varvec{C}}}\right)-100$$

where: “***mean S***” is the mean algal concentration in samples, and “***mean C***” is the mean algal concentration in control.

As performed for *R. subcapitata* assay (“***Raphidocelis subcapitata – growth inhibition***” section), samples which showed a ΔG% > 40% (biostimulation) in respect to control (Table B — Supplementary information) have been retested at concentrations from 1 to 100 mg L^−1^. Preparation of sample concentrations followed the same procedure of *R. subcapitata*.

Potassium dichromate was used as reference toxicant for this species — EC_50_ of 7.43 mg L^−1^ (6.82–8.24). For statistical analysis, a Student’s *t*-test was performed between (1) each concentration of unadj-pH and control; (2) adj-pH and control and (3) each concentration of unadj-pH and adj-pH.

#### Ficopomatus enigmaticus — larval development assay

*F*. *enigmaticus* reef pieces were collected in S. Rossore-Migliarino Regional Park-Fiume Morto (Pisa, Italy), in the late summer period (September), and transferred to the laboratory under a wet cloth (embedded with water from the site). The same water, from the sampling site, was used to do the maintenance aquarium setup. Environmental salinity was 21. In laboratory, salinity was increased up to a maximum of 3 points/day, by adding fresh ASW (S = 40), until reaching 30, value defined as within the optimum range for *F*. *enigmaticus* larval development (Oliva et al. 2018). For 7 days, needed for acclimation, organisms were daily fed with *Isochrysis galbana* algal suspension (10^4^ cells mL^−1^). At the end of those 7 days, conditions in the aquaria were considered viable for the bioassay execution (T 22 ± 1 °C, oxygen saturation > 90%, salinity 30, pH 8.1 ± 0.1, photoperiod — 10 h light:14 h darkness). The assay integrally followed the methodology of Oliva et al. (2019). Trocophore larvae were exposed to 90% of maximum concentration samples (100 mg L^−1^) for 48 h. Three replicates per toxicant were kept at 25 ± 2 °C, under a 14 h light:10 h darkness photoperiod (1000–2000 lx). Finished the test, the next step was to calculate the mean percentage of badly developed larvae with and without correction (plus the standard deviation) by the Abbott’s $\mathrm{formula}:\frac{\left(mean\;sample-mean\;CTRL\right)}{\left(100-mean\;CTRL\right)}\times100$.

The reference toxicant was copper sulphate pentahydrate, with an EC_50_ of 48.56 mg L^−1^ (44.95–51.99).

## Results

### Daphnia magna — immobilization

Table 2 shows the results obtained for the *D*. *magna* acute ecotoxicity assay. The highest immobilization percentage, although a low value (− 15%), was observed after exposure to choline acetate:imidazole. In descending order, three NADESs have induced immobilization reaching the 10%, them being betaine:levulinic acid, cholinium bitartrate:citric acid and choline acetate:diglycolic acid. The remaining eleven compounds displayed even lower or null immobilization percentages, from 0 to a maximum of 5%. NADESs’ toxicity towards *D*. *magna* was pretty much negligible.

**Table 2:** Percentage ± standard deviation of immobile *D. magna* individuals after 48 h of exposure to maximum concentration samples (100 mg/L). The assay was performed at 25° ± 2° C, in darkness. N°. organisms/replicate = 10; n = 3. EC_50_ K_2_Cr_2_O_7_ = 0.586 mg/L (C.L. 95% = 0.401–0.772)

Samples	Mean immobile individual % at max concentration	S.D
CTRL	0.00	0.00
betaine:ethylene glycol	5.00	0.50
betaine:citric acid	0.00	0.00
betaine:glycerol	5.00	0.50
betaine:levulinic acid	10.00	0.58
betaine:L-lactic acid	0.00	0.00
proline:glycerol	5.00	0.50
proline:lactic acid	0.00	0.00
proline:levulinic acid	0.00	0.00
proline:malic acid	0.00	0.00
cholinium bitartrate:citric acid	10.00	0.58
choline acetate:imidazole	15.00	0.50
choline acetate:levulinic acid	5.00	0.50
choline acetate:glycolic acid	0.00	0.00
choline acetate:diglycolic acid	10.00	0.58
choline acetate:citric acid	5.00	0.50

### Raphidocelis subcapitata — growth inhibition

**Fig. 1:**
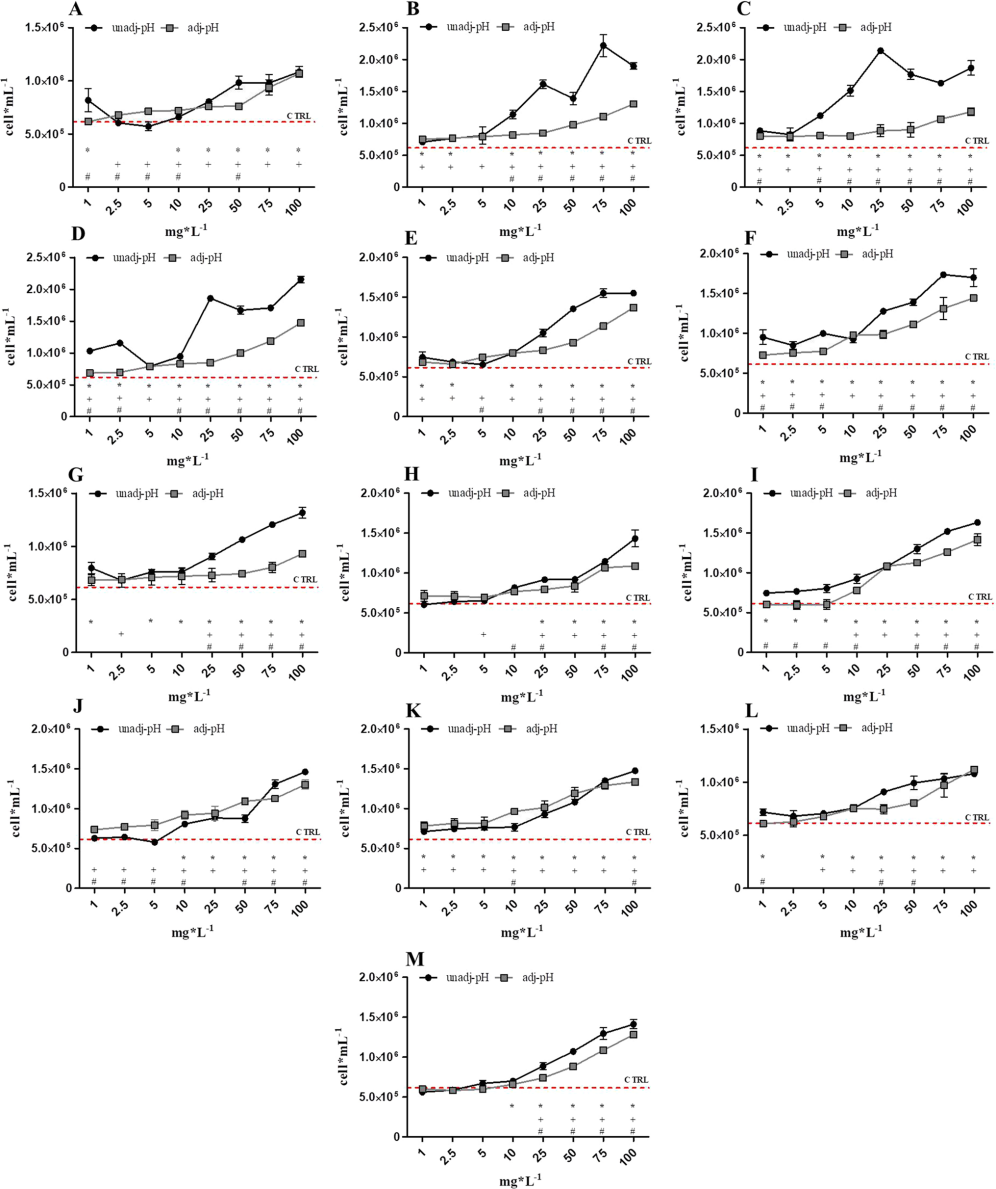
Concentration–response graphs of samples with ΔG% > 40% after *R. subcapitata* inhibition of growth assay with both pH-unadjusted (unadj-pH) and adjusted (adj-pH) samples. Red line is the mean algal concentration in controls. (**A)** betaine:ethylene glycol; (**B**) betaine:citric acid; (**C**) betaine:glycerol; (**D**) betaine:L-lactic acid; (**E**) proline:glycerol; (**F**) proline:lactic acid; (**G**) proline:levulinic acid; (**H**) cholinium bitartrate:citric acid; (**I**) proline:malic acid; (**J**) choline acetate:levulinic acid; (**K**) choline acetate:glycolic acid; (**L**) choline acetate:diglycolic acid; (**M**) choline acetate:citric acid. Results are expressed as mean algal concentration (cells mL^−1^) ± standard deviation (n = 3). A Student’s *t*-test was performed between each concentration of unadj-pH and control (*), between adj-pH and control ( +) and between unadj-pH and adj-pH (#). *, + , # = statistically significant difference, *p* < 0.05

Figure 1 represents the growth inhibition behaviour of *R*. *subcapitata* algae exposed to different concentrations of thirteen NADESs. These thirteen NADESs — betaine:ethylene glycol, betaine:citric acid, betaine:glycerol, betaine:L-lactic acid, proline:glycerol, proline:lactic acid, proline:levulinic acid, cholinium bitartrate:citric acid, proline:malic acid, choline acetate:levulinic acid, choline acetate:glycolic acid, choline acetate:diglycolic acid and choline acetate:citric acid — produced a biostimulation > 40% in respect to control (Table A — Supplementary information) and, thus, have been retested at concentrations of 1 to 100 mg L^−1^ with both pH uncorrected and corrected to control values. In all cases, the concentration–response curve showed a trend of increasing algal growth with increasing concentrations of all thirteen compounds. In contaminated media, algal concentrations were always superior to the value registered for the control (red line). Regarding differences between algal growth registered on unadj-pH and adj-pH samples, a reduction in algal concentration was generally observed for all adj-pH samples. The concentration–response trend, however, remained similar between the two sets of samples.

### Aliivibrio fischeri — inhibition of bioluminescence

Table 3 displays the results obtained for the bioluminescence inhibition test with *A*. *fischeri* in fresh and marine water, respectively. Following the freshwater protocol, differences between bioluminescence percentage of controls and samples gave high negative values, which indicated a stimulation of bioluminescence. Betaine:ethylene glycol was the NADESs producing the highest bioluminescence stimulation (− 22.24, at 15 min of exposure). On the other hand, for the same compounds but in marine water, a slight inhibition of bioluminescence was observed, represented by low and positive values. Betaine:L-lactic acid was the NADESs inducing the highest degree of bioluminescence inhibition (+ 0.03, at 15 min of exposure).

**Table 3:** Inhibition of bioluminescence (I%) of *A. fischeri* after 15 and 30′ of exposure to 90% of maximum concentration samples (100 mg/L). The assay was performed at 15° ± 1 °C. Marine protocol EC50 Zn^2+^  = 10.26 mg/L (C.L. 95% = 9.22–11.43); Freshwater protocol EC50 Zn^2+^  = 1.04 mg/L (C.L. 95% = 0.80–1.36)

Samples	Bioluminescence inhibition at max concentration							
	Marine protocol				Freshwater protocol			
	Mean I% (15′)	S.D	Mean I% (30′)	S.D	Mean I% (15′)	S.D	Mean I% (30′)	S.D
betaine:ethylene glycol	6.16	0.32	4.80	0.62	− 22.24	0.13	− 15.72	1.56
betaine:citric acid	7.08	1.69	7.79	2.54	− 8.59	0.85	− 14.01	0.91
betaine:glycerol	3.79	0.92	4.33	0.17	− 10.19	3.97	− 13.95	0.09
betaine:levulinic acid	4.68	1.91	3.82	1.20	− 12.82	1.80	− 14.93	0.21
betaine:L-lactic acid	0.03	1.11	0.51	0.53	− 13.84	3.82	− 19.12	6.02
proline:glycerol	2.76	1.91	2.89	2.17	− 11.57	4.68	− 16.76	7.08
proline:lactic acid	− 0.26	0.89	− 1.09	0.51	− 14.53	1.65	− 17.38	0.62
proline:levulinic acid	3.78	2.42	3.43	0.02	− 7.97	2.80	− 14.58	2.00
proline:malic acid	11.15	1.26	23.45	0.84	− 5.28	2.97	− 6.82	2.18
cholinium bitartrate:citric acid	6.41	1.81	10.51	0.71	− 14.71	2.84	− 17.17	4.84
choline acetate:imidazole	4.21	1.43	5.51	0.09	− 13.25	0.52	− 14.26	1.56
choline acetate:levulinic acid	6.89	0.17	9.79	0.83	− 10.42	0.40	− 13.87	0.08
choline acetate:glycolic acid	1.20	2.25	4.65	0.95	− 10.87	1.04	− 15.25	1.31
choline acetate:diglycolic acid	2.80	0.52	3.38	0.32	− 15.43	1.56	− 17.94	1.47
choline acetate:citric acid	5.95	2.44	11.77	0.43	− 11.03	0.54	− 14.61	0.33

### Phaeodactylum tricornutum — growth inhibition

**Fig. 2:**
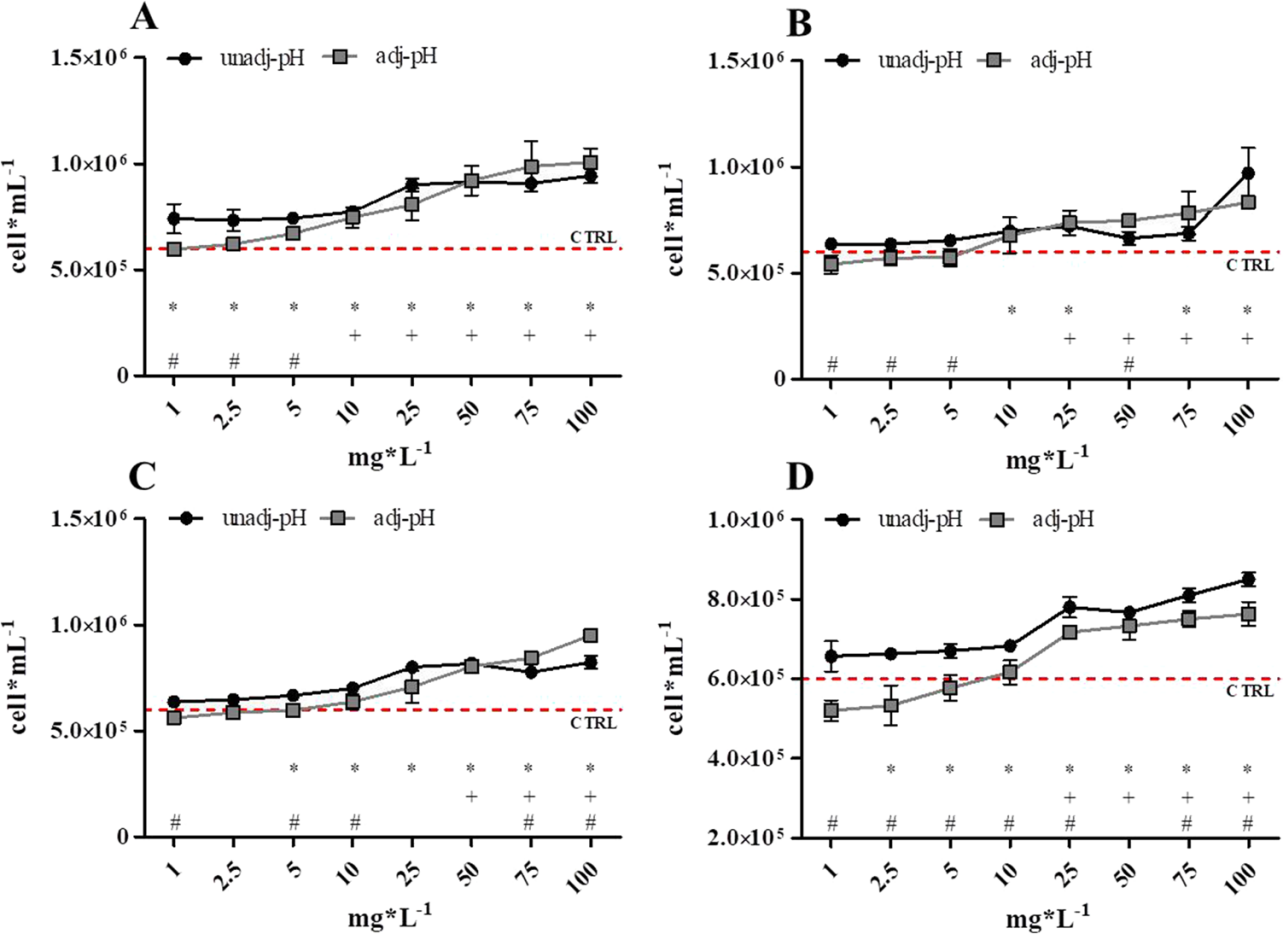
Concentration–response graphs of samples with ΔG% > 40% after *P. tricornutum* inhibition of growth assay with both pH-unadjusted (unadj-pH) and adjusted (adj-pH) samples. Red line is the mean algal concentration in controls. (**A**) betaine:L-lactic acid; (**B**) proline:glycerol; (**C**) proline:L-lactic acid; (**D**) proline:malic acid. Results are expressed as mean algal concentration (cells mL.^−1^) ± standard deviation (n = 3). A Student’s *t*-test was performed between each concentration of unadj-pH and control (*), between adj-pH and control ( +) and between unadj-pH and adj-pH (#). *, + , # = statistically significant difference, *p* < 0.05

Figure 2 illustrates the growth inhibition response of *P*. *tricornutum* algae exposed to eight concentrations of four NADESs: betaine:L-lactic acid, proline:glycerol, proline:L-lactic acid and proline:malic acid. Once again, biostimulation was considered as an effect and only four compounds have shown growth percentage differences > 40%, having been selected for further testing (Table B — Supplementary information). Concentration–response curves exhibited an increase in algal cells concentration with increasing loads of four of the assessed NADESs. This trend was more noticeable in the case of proline-based NADESs, which induced important biostimulation in this species. Regarding differences between algal growth registered on unadj-pH and adj-pH samples, in the case of *P. tricornutum* assay, they were generally less evident than *R. subcapitata*, with the exception of the sample proline:malic acid. This showed a reduction in algal concentrations at all tested concentration, maintaining, again, the same tendency.

### Ficopomatus enigmaticus — larval development assay

Table 4 presents the mean values of badly developed larvae obtained for the bioassay with *F*. *enigmaticus*. As has happened for the species mentioned above, tested NADESs have produced very low levels of toxicity. The two compounds inducing the highest percentages of malformation were choline acetate:citric acid and choline:acetate imidazole, with values of 27.00% and 30.67%, respectively. Betaine:L-lactic acid was the NADESs which showed the least effect (8.67%).

**Table 4:** Inhibition of larval development with *F. enigmaticus* after 48 h of exposure to 90% of maximum concentration samples (100 mg/L). The assay was performed at 25° ± 2° C, under a 14 h light:10 h darkness photoperiod (1000–2000 lx); n = 3. In the table, both the mean percentage of badly developed larvae without and with correction with Abbott’s formula, and the standard deviation, are reported. EC50 Cu^2+^  = 48.56 mg/L (C.L. 95% = 44.95–51.99)

Samples	Mean of bad developed larvae at max concentration	Abbott Correction	S.D
CTRL	13.00	0.00	4.36
betaine:ethylene glycol	17.33	4.98	9.45
betaine:citric acid	21.33	9.58	1.15
betaine:glycerol	18.33	6.13	2.52
betaine:levulinic acid	20.33	8.43	8.50
betaine:L-lactic acid	8.67	− 4.98	3.06
proline:glycerol	11.00	− 2.30	1.00
proline:lactic acid	9.67	− 3.83	3.21
proline:levulinic acid	17.00	4.60	2.65
proline:malic acid	11.67	− 1.53	2.52
cholinium bitartrate:citric acid	23.00	11.49	7.55
choline acetate:imidazole	30.67	20.31	7.51
choline acetate:levulinic acid	14.67	1.92	4.16
choline acetate:glycolic acid	15.33	2.68	3.06
choline acetate:diglycolic acid	18.67	6.51	5.77
choline acetate:citric acid	27.00	16.09	4.36

## Discussion

Due to the diffused claim that DESs are clean solvents, liable to substitute in-use toxic ones, the present study analysed the exposure effects of 15 selected NADESs on the freshwater species *D*. *magna* (immobilization) and *R*. *subcapitata* (growth inhibition), as well as the marine ones *P*. *tricornutum* (growth inhibition), *F*. *enigmaticus* (larval development success) and *A*. *fischeri* (bioluminescence inhibition). Although these 15 NADESs did not induce toxic effects in terms of selected endpoints on any of these species, an environmental issue would be likely to arise from the presence of this kind of compounds in aquatic ecosystems.

In agreement with the supposed environmental sustainability of NADESs, none of the tested combinations have induced significant toxicity towards *D*. *magna* and *F*. *enigmaticus*. According to Lapeña et al. (2021), water as chemical solvent can interfere with the structure of DESs. When hydrogen bonding is established between water molecules and DESs’ components, it is possible that the DESs’ structural complex may be disrupted. In fact, most diffused DESs are hydrophilic (Singh et al. 2021) and, according to Morais et al. (2015), it is possible to assume that DESs may pass the cellular membrane and disrupt cytoplasmic anion pool, altering the pH equilibrium by the action of their acidic portion. However, considering that these two bioassays were set in aqueous media (fresh and marine water) the observed lack of effect might be related to the water-mediated structural disruption and consequent exposure to DESs non-toxic initial components. In addition, DESs with water within their structure might display higher fluidity (Lapeña et al. 2021), becoming non-threatening for organisms’ mobility (*D*. *magna*) or development (*F. enigmaticus* larvae). Considering the aforementioned, such a low degree of toxicity would be likely attributable to assay conditions and/or genetic determinism, not directly related with DESs’ mode of action. However, it may be of interest to compare findings of the present work with those by Samorì et al. (2010), where the authors assessed several *N*-methylimidazolium-based ionic liquids (ILs) with *D. magna* and *A. fischeri* bioassays. What emerged was a significant immobilization effect on *D. magna* of some tested ILs. In the present work, the NADES with imidazole as a component, so structurally comparable to *N*-methylimidazolium-based ILs, showed the highest toxic effect against *F. enigmaticus*; however, it was not high enough to calculate ecotoxicological parameters such as EC_50._ What we can hypothesize is a putative differential effect of ionic bond, present on ILs, in respect to hydrogen bond of NADES in maintaining structural features, related to toxic effect of the compounds.

In addition to previous observations, it is important to underline that evaluated concentrations were low when compared to ranges of 150–12,000 mg L^−1^ used by Lapeña et al. (2021), for *D*. *magna*. Nevertheless, Macário et al. (2018) and Lapeña et al. (2021) have observed similar results to the ones of the present study, which would also be the first to report data obtained from a polychaete species after exposure to NADESs. Such evidence might reinforce the sustainable character and poor toxic profile of NADESs.

Concerning *A*. *fischeri*, the low effect observed with the marine water protocol may be related to putative interactions between salt water loaded with sodium and chloride and DESs’ charged parts, resulting in a reduction of DESs’ permeability properties with consequent loss of toxic ability (Latała et al. 2005). Regarding the same species, freshwater protocol results underlined a slight biostimulation which, despite being lower than 20% for all assessed compounds, indicate a clear difference from the same samples tested with the marine protocol. Given that *A*. *fischeri* is a marine bacterium, it sounds plausible that chemical interactions between DESs and the respective aqueous media may differ dependently by ionic composition of exposure water. The pattern of responses observed with both *A. fischeri* assays was even emphasized by the two algal tests performed in this study. Indeed, both *R*. *subcapitata* (freshwater) and *P*. *tricornutum* (marine water) growth resulted to be not inhibited by any tested compound, but highly biostimulated by several NADESs. In particular, *P. tricornutum* growth was significantly stimulated by six different NADES, of which four showed a ΔG% (see Tables A and B in the Supplementary information) higher than 40% in respect to controls, while *R. subcapitata* growth was stimulated by thirteen samples, of which five with ΔG% higher than 40% and eight with ΔG% higher than 100% in respect to controls. No other studies reported algal biostimulation as a result of exposure to NADESs; however, as stated in the new Italian Ministerial Decree (D.M. 173: 2016), algal biostimulation can be considered as negative effect, as well as growth inhibition. As expected, the observed effects in marine algae were lower than those in freshwater algae. According with findings of Latała et al. (2005) for ionic liquids, we can hypothesise that the reduction of the registered effects in marine algae may be due to the interaction of salt water chloride anions with DESs’ charged parts. Further, the observed biostimulation may likely be related with cellular assimilation of the DESs’ natural and non-toxic starting materials which, dispersed in the culture medium, would play the role of phytonutrients. As an example, choline, also known as vitamin B_4_, is an essential nutrient for living organisms (Zeisel and da Costa 2009; Yang 2019). Moreover, this algal growth stimulation can be linked to metabolic requirements of important nutrients for cellular function which usually display low toxicity and which are common components of NADES (Hayyan et al. 2016; Mbous et al. 2017; Radošević et al. 2018). pH is also considered another factor affecting algal abundance. Low pH values (from 6.6 to 5.0) are considered as a booster for algal growth (Leavitt et al. 1999). Accordantly, our results showed a more evident effect of NADES in inducing algal growth if tested with unmodified pH, which were generally lower than 7.5. Moreover, differences between the two algal species behaviour were observed. *R. subcapitata* showed a lower tolerance to pH variations than *P. tricornutum*, confirming how different species of algae have tolerances for different pH ranges (Bergstrom et al. 2007).

Regarding pH effect, Giner et al. (2020) recently proposed a QSAR model for NADES toxicological evaluations on *A. fischeri* using mixing rules to include any composition of different components. Among their observations, the authors underlined a positive correlation between the toxic effects towards *A. fischeri*, in terms of EC_50_, and the presence of an organic acid in the mixture, indicating low pH values of tested samples and relevant for negative biological effects on this species. That observation is aligned on those reported in the present work for microalgae, indicating the relevancy of the parameter “pH” when assessing NADES toxicity.

Considering an ever-growing interest in NADESs, associated to their wide applicability, designability, cheapness and biocompatibility (Paiva et al. 2014; Yang 2018), it becomes expected that, in a near future, they will end up in natural water bodies, in ever-increasing concentrations. However, basing on present results in which a significant algal growth was observable at relatively low concentrations (1–100 mg L^−1^), a serious environmental threat may come from the phenomenon of eutrophication. In particular, this condition implicates oxygen depletion and resident biota alterations due to excess of nutrients and consequent intensification of primary producer activity (Wurtsbaugh et al. 2019). This occurrence may affect lake environments more than coastal ones, as the water exchange is more limited and, thus, oxygen renewal does not keep up, added with lower pH and buffering features of freshwater compared to marine water.

Despite the fact that NADESs may induce negligible toxicity to invertebrate species, with obtained data encouraging their labelling as “sustainable” solvents, the liability of their intentional or accidental release into aquatic systems will likely pose a serious risk in terms of ecosystem functioning impairments. The detected algal biostimulation by several NADESs can pose risk to aquatic environments in terms of additive inputs to other factors influencing the impairment of aquatic ecosystems such as nutrient over-enrichment and climate change (namely acidification and temperature increase). For this reason the future potential immission of these solvents in aquatic environments has to be carefully monitored.

## Conclusions

Obtained results indicated that the selected NADESs were non-toxic for *D*. *magna*, *F*. *enigmaticus* and *A. fischeri*. However, aquatic algae growth was highly biostimulated in the presence of these compounds. Besides the assessing of a wide set of aquatic invertebrate species, which is still lacking in the literature, this study reveals that direct ecotoxicity evaluations throughout single/bioassay-batch testing might not be enough to assure and validate the labelling of a certain chemical as “green”, “clean” or “environmentally friendly”. A suggestion for further works and investigations could be the complement of ecotoxicological bioassays with an embracing approach. In fact, a natural system can be influenced by both biotic and abiotic factors and may synergistically act in the presence of other chemical compounds such as NADESs/DESs. Monitoring biological effects of these compounds could play a key role since PECs (predicted environmental concentrations) are expected to arise and increase in the near future due to an extensive use in industrial processes.

## Supplementary Information

Below is the link to the electronic supplementary material.

Supplementary file1 (DOCX 25 KB)

## Data Availability

All data generated or analysed during this study are included in this published article.
