# Supplementary material for: Are natural deep eutectic solvents always a sustainable option? A bioassay-based study
Source: Environ Sci Pollut Res Int. 2022 Oct 4;30(7):17268–79. doi: 10.1007/s11356-022-23362-5 (PMC9928812; doi:10.1007/s11356-022-23362-5)
Supplement: Supplementary file 1 — Supplementary file1 (DOCX 25 KB) [file 11356_2022_23362_MOESM1_ESM.docx]

Table A. Inhibition of growth of *R. subcapitata* after 72h of exposure to maximum concentration samples (100 mg/L). The assay was performed at 20°±2° C, under continuous illumination (6000-8000 lx). n = 3. ΔG % = growth percentage difference (as cells mL^-1^) with control. EC50 K_2_Cr_2_O_7_ = 0.742 mg/L (C.L. 95% = 0.648-0.808).

|  | **pH**  **(at maximum concentration)** | **Algal density at max concentration (Cells mL^-1^)** | | **ΔG %** | **Effect** |
| --- | --- | --- | --- | --- | --- |
|  |  | **Mean** | **S.D.** |  |  |
| **CTRL** | 8.10 | 520833 | 14434 | - | - |
| **betaine:ethylene glycol** | 7.85 | 1025000 | 25000 | 96.8 | Biostimulation (>40%) |
| **betaine:citric acid** | 6.08 | 1066667 | 52042 | 104.8 | Biostimulation (>100%) |
| **betaine:glycerol** | 7.04 | 979167 | 31458 | 88 | Biostimulation (>40%) |
| **betaine:levulinic acid** | 6.24 | 600000 | 21651 | 15.2 | n.d. |
| **betaine:L-lactic acid** | 5.81 | 1387500 | 33072 | 166.4 | Biostimulation (>100%) |
| **proline:glycerol** | 7.81 | 1233333 | 19094 | 136.8 | Biostimulation (>100%) |
| **proline:lactic acid** | 5.63 | 1800000 | 37500 | 245.6 | Biostimulation (>100%) |
| **proline:levulinic acid** | 6.24 | 991667 | 31458 | 90.4 | Biostimulation (>40%) |
| **cholinium bitartrate:citric acid** | 5.76 | 929167 | 75346 | 78.4 | Biostimulation (>40%) |
| **proline:malic acid** | 5.47 | 1595833 | 36084 | 206.4 | Biostimulation (>100%) |
| **choline acetate:imidazole** | 7.58 | 587500 | 37500 | 12.8 | n.d. |
| **choline acetate:levulinic acid** | 6.97 | 1070833 | 36084 | 105.6 | Biostimulation (>100%) |
| **choline acetate:glycolic acid** | 6.75 | 850000 | 54486 | 63.2 | Biostimulation (>40%) |
| **choline acetate:diglycolic acid** | 5.72 | 1079167 | 61661 | 107.2 | Biostimulation (>100%) |
| **choline acetate:citric acid** | 6.33 | 1137500 | 66144 | 118.4 | Biostimulation (>100%) |

Table B. Inhibition of growth of *P. tricornutum* after 72h of exposure to maximum concentration samples (100 mg/L). The assay was performed at 20°±2° C, under continuous illumination (6000-8000 lx); n = 3. ΔG % = growth percentage difference (as cells mL^-1^) with control. EC50 K_2_Cr_2_O_7_ = 7.43 mg/L (C.L. 95% = 6.82-8.24).

|  | **pH**  **(at maximum concentration)** | **Algal density at max concentration (Cells*mL^-1^)** | | **ΔG %** | **Effect** |
| --- | --- | --- | --- | --- | --- |
|  |  | **Mean** | **S.D.** |  |  |
| **CTRL** | 8.10 | 5500000 | 360555 | - | - |
| **betaine:ethylene glycol** | 8.08 | 5600000 | 360555 | 1.82 | n.d. |
| **betaine:citric acid** | 7.17 | 6866667 | 611010 | 24.85 | Biostimulation (<40%) |
| **betaine:glycerol** | 7.54 | 6233333 | 321455 | 13.33 | n.d. |
| **betaine:levulinic acid** | 7.33 | 5933333 | 208167 | 7.88 | n.d. |
| **betaine:L-lactic acid** | 6.94 | 7833333 | 351188 | 42.42 | Biostimulation (>40%) |
| **proline:glycerol** | 7.92 | 10033333 | 450925 | 82.42 | Biostimulation (>40%) |
| **proline:lactic acid** | 6.84 | 10866667 | 152753 | 97.58 | Biostimulation (>40%) |
| **proline:levulinic acid** | 7.23 | 7133333 | 404145 | 29.7 | Biostimulation (<40%) |
| **cholinium bitartrate:citric acid** | 8.11 | 6000000 | 346410 | 9.09 | n.d. |
| **proline:malic acid** | 7.65 | 8433333 | 251661 | 53.33 | Biostimulation (>40%) |
| **choline acetate:imidazole** | 7.83 | 5600000 | 200000 | 1.82 | n.d. |
| **choline acetate:levulinic acid** | 7.97 | 5600000 | 346410 | 1.82 | n.d. |
| **choline acetate:glycolic acid** | 7.95 | 5900000 | 264575 | 7.27 | n.d. |
| **choline acetate:diglycolic acid** | 7.82 | 6133333 | 57735 | 11.52 | n.d. |
| **choline acetate:citric acid** | 8.00 | 6500000 | 100000 | 18.18 | n.d. |
